# Retraction Note: Long non-coding RNA GATA6-AS1 upregulates GATA6 to regulate the biological behaviors of lung adenocarcinoma cells

**DOI:** 10.1186/s12890-022-02142-4

**Published:** 2022-09-14

**Authors:** Honggang Kang, Dan Ma, Jing Zhang, Jun Zhao, Mengxiang Yang

**Affiliations:** Department of Oncology, Liaocheng People’s Hospital, 67 Dongchang West Road, Liaocheng, 252000 Shandong China

## Retraction Note to: BMC Pulm Med (2021) 21:166 10.1186/s12890-021-01521-7

The authors have retracted this article because of problems with Figure 4 and Figure 1, namely:

- Figure 4H (panel H1975 vs pcDNA3.1-NC) overlaps with Figure 1F (panel A549 vs sh-NC) in a different article [1]
- Figure 4H (panel H1975 vs pcDNA3.1-NC) overlaps with Figure 5C (panel CAL27, miR-106b-5p mimics + pcDNA3.1/DAB2) in a different article [2]
- Figure 1F overlaps with Figure 5b in a different article [3]

The authors have stated that some of the cell biology experiments were performed by external companies. All authors agree with this retraction.
